# Analysis of factors influencing molecular testing at diagnostic of colorectal cancer

**DOI:** 10.1186/s12885-017-3759-6

**Published:** 2017-11-14

**Authors:** Quentin Thiebault, Gautier Defossez, Lucie Karayan-Tapon, Pierre Ingrand, Christine Silvain, David Tougeron

**Affiliations:** 1 Department of Gastroenterology, Poitiers University Hospital, 2 rue de la Milétrie, 86000 Poitiers Cedex, France; 2 Poitou-Charentes General Cancer Registry, Poitiers University Hospital, University of Poitiers, Poitiers, France; 3 INSERM, CIC 1402, Poitiers, France; 4 Department of Cancer Biology, Poitiers University Hospital, Poitiers, France; 5 Laboratory Inflammation, Tissus Epithéliaux et Cytokines, EA 4331, University of Poitiers, Poitiers, France

**Keywords:** Colorectal cancer, *KRAS*, Mutation, Molecular testing, *BRAF*, Microsatellite instability

## Abstract

**Background:**

The aim of the study was to evaluate the current rate of molecular testing prescription (*KRAS* codons 12/13, *BRAF* and microsatellite instability (MSI)) in newly diagnosed colorectal cancer (CRC) patients and to determine which factors influence testing.

**Methods:**

All incident CRC cases in 2010 were identified in the Poitou-Charentes General Cancer Registry. The exhaustive molecular testing performed was accessed in the French molecular genetics platform. Factors influencing prescription were analyzed using logistic regression.

**Results:**

Among the 1269 CRCs included in the study, *KRAS*, *BRAF* and MSI testing accounted for 35.1%, 10.5% and 10.9%, respectively. *KRAS* testing was carried out in 65.5% of metastatic CRCs, and 26.1% of non-metastatic CRCs. Among metastatic CRCs, age (<60 years), site of primary tumour (left colon) and geographical area of treatment were factors related to *KRAS* testing. *BRAF* testing was contemporary to *KRAS* testing for 92.5% of patients. Factors related to MSI testing were age (<60 years), TNM stage (stage IV) and geographical area of treatment. Among CRC patients under 60 years old, only 37.5% had MSI testing.

**Conclusion:**

These results underscore the need to reduce disparities in CRC molecular testing and highlight the limited application of the French guidelines, especially concerning MSI testing.

## Background

Colorectal cancer (CRC) is the third most common cancer worldwide [1]. To date, colorectal carcinogenesis has been classified in three distinct pathways: chromosomal instability (85%), microsatellite instability (MSI) (15%) and CpG island methylator phenotype (25%). MSI is related to a deficient DNA mismatch repair (dMMR) system due to germline mutation in a MMR gene in Lynch syndrome (LS), or more commonly to an epigenetic inactivation of *MLH1* in sporadic cases. Approximately 45% of CRC cases have a *KRAS* mutation [2] and only patients with wild-type (WT) *RAS* metastatic CRC (mCRC) may benefit from anti-epidermal growth factor receptor monoclonal antibody therapy (anti-EGFR mAbs) [3, 4]. A *BRAF* mutation (V600E) is present in approximately 12% of CRCs and confers a poor prognosis, especially in mCRCs [5–8]. In dMMR CRC, *BRAF* mutation is specific to a sporadic origin and eliminates a LS.

Since 2006, the French National Cancer Institute (INCa) has been supporting a national network of 28 hospital molecular genetics platforms throughout France, offering patients all essential molecular genetics techniques for all cancers. For CRC, *KRAS* (now complete *RAS*), *BRAF* and MSI testing are routinely performed. Since 2008, *KRAS* testing is supposed to be performed in all mCRC cases. Since *KRAS* and *BRAF* mutations are mutually exclusive [8], *BRAF* testing is performed only in *KRAS* WT tumours. In France, MSI testing is recommended in patients with a CRC at an age lower than 60 and/or if family history suggests a LS. Nevertheless, epidemiological data concerning these different testing procedures are lacking. A recent French retrospective study revealed that 81.1% of patients with a mCRC had *KRAS* testing [9]. This study has some limitations due the non-exhaustiveness of incident CRC cases included and patient recruitment based on physician willingness. The General Cancer Registry in the Poitou-Charentes region (GCRPC) covers an administrative region of 1.8 million people in south-western France (available at http://medphar.univ-poitiers.fr/registre-cancers-poitou-charentes/) and has been collecting all incident cancer cases, thereby enabling exhaustive analysis of the molecular analyses (using INCa molecular cancer genetics platform) performed in all incident CRC cases. The aim of the study was to analyze routine practice of *KRAS*, *BRAF* and MSI molecular testing among all the CRC patients in Poitou-Charentes diagnosed in 2010.

## Methods

### Study population

**Fig. 1:**
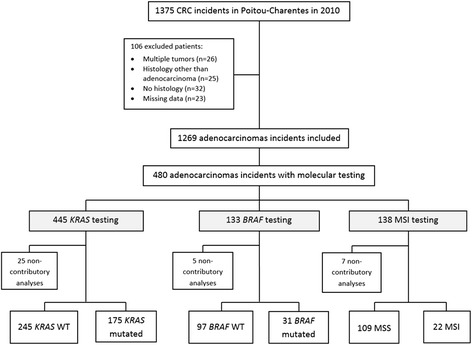
Flowchart of the study. Abbreviations: CRC, colorectal cancer; WT, wild-type; MSI, microsatellite instability; MSS, microsatellite stable

Since 2008, the GCRPC has included all incident cases of cancer, involving subjects regularly residing in the Poitou-Charentes region at the time of diagnosis, whatever the place of care. The Poitou-Charentes region comprises four departments: Charente, Charente-Maritime, Deux-Sèvres and Vienne. The minimum items recorded in the GCRPC were demographic data, tumour characteristics and treatment. According to the French law the data collected from the GCRPC was approved by the CCTIRS (Comité Consultatif sur le Traitement de l’Information en matière de Recherche dans le domaine de la Santé, approval n°07–374) and the CNIL (Commission Nationale de l’Informatique et des Libertés, approval n°907,303). Using the GCRPC 1375 incident CRC patients were identified in 2010 and after exclusion of non-relevant cases, 1269 patients were included in the study (Fig. 1).

### Molecular testing

In 2010, *KRAS* mutational status (exon 2 codons 12 and 13) was determined at the specific request of a clinician. Regarding *BRAF* mutational status (V600E), analysis was mostly performed by the INCa hospital molecular genetics platforms in case of *KRAS* wild-type status. MSI was to be determined at the specific request of the clinician (suspicion of LS) or by the platforms for patients under 60 years old. All of the exhaustive molecular analyses (*n* = 480) from the different hospital molecular genetics platforms were itemized (Poitiers (*n* = 401) and other platforms (*n* = 79)).

### Statistical analysis

The aim of the study was to evaluate the rate of prescription of molecular testing (*KRAS*, *BRAF* and MSI) regarding guidelines applicable in 2010 to newly diagnosed CRC patients. Secondary objectives were to analyze which criteria influenced *KRAS* molecular testing for metastatic and non-metastatic CRC patients respectively, and which characteristics influenced MSI molecular testing for all CRC patients.

The study was conducted in accordance with the Strengthening the Reporting of Observational Studies in Epidemiology (STROBE) statement. The descriptive statistics used for quantitative parameters were mean and standard deviation; for qualitative parameters were frequency and percentage. A logistic regression was carried out on factors that could promote *KRAS* testing and MSI testing and determined odds ratios (OR) with a 95% confidence interval (CI). The geographical area of primary treatment was defined from the location of the center where the first treatment of CRC was performed. Status of the center was categorized as public, private or university hospital.

Statistically significant factors derived from univariate analysis (*P* values <0.25) were selected for multivariate analysis using a stepwise descending selection procedure with a significance threshold at 0.05. Possible interactions between independent risk factors were tested by including proper cross-product terms in the regression models, and likelihood ratio tests comparing models with and without the interaction term were used to estimate the significance of the interaction. Data management and statistical analyses were performed using SAS software version 9.4 (SAS Institute, Cary, NC, USA).

## Results

### Population

Between January 1st and December 31st 2010, 1269 incident cases of CRC were included in the study. The age-standardized incidence rates of CRC were respectively 38.3 per 100,000 in men and 26.9 per 100,000 in women. Mean age was 71.9 ± 11.8 years (Table 1). At diagnosis, 22.8% of CRCs were metastatic and 77.2% were non-metastatic.

**Table 1:** Patient and tumour characteristics

	All patients (*n* = 1269)	Patients without molecular test^a^ (*n* = 789)	Patients with at least one molecular test^a^ (*n* = 480)
Age (years, SD)	71.9 ± 11.8	74.4 ± 11.3	67.8 ± 11.4
Sex			
Women	578 (45.5%)	356 (45.1%)	222 (46.3%)
Men	691 (54.5%)	433 (54.9%)	258 (53.8%)
Site of the primary tumour			
Rectum	309 (24.4%)	220 (28.0%)	89 (18.8%)
Right colon	437 (34.4%)	268 (34.0%)	169 (35.0%)
Left colon	523 (41.2%)	301 (38.0%)	222 (46.3%)
TNM stage			
Stage I	219 (17.3%)	194 (24.6%)	25 (5.2%)
Stage II	380 (29.9%)	265 (33.6%)	115 (24.0%)
Stage III	380 (29.9%)	232 (29.4%)	148 (30.8%)
Stage IV	290 (22.8%)	98 (12.4%)	192 (40.0%)
Tumour grade (MD = 173)			
Well	436 (39.8%)	250 (37.7%)	186 (43.3%)
Moderate	576 (52.5%)	369 (55.4%)	207 (47.9%)
Poor	84 (7.7%)	46 (6.9%)	38 (8.8%)
Geographical area of primary treatment (MD = 4)			
Charente-Maritime	392 (31.0%)	286 (36.4%)	106 (22.1%)
Charente	226 (17.9%)	171 (21.6%)	55 (11.5%)
Deux-Sèvres	227 (17.9%)	110 (14.1%)	117 (24.4%)
Vienne	301 (23.8%)	137 (17.4%)	164 (34.2%)
Outside the region	119 (9.4%)	82 (10.4%)	37 (7.7%)
Status of the center (MD = 4)			
Public Hospital	551 (43.5%)	381 (48.6%)	170 (35.5%)
Private hospital	512 (40.5%)	292 (37.2%)	220 (45.7%)
University hospital	202 (16.0%)	112 (14.3%)	90 (18.8%)

*MD* missing data, *SD* standard deviation

^a^Molecular test defined as *KRAS*, *BRAF* and/or MSI testing

### Molecular testing

Overall, 480 CRCs (37.8% of the cohort) had at least one molecular test (*KRAS, BRAF* or MSI). *KRAS* was mutated in 41.7% of cases (*n* = 175/420), *BRAF* mutation in 24.2% (*n* = 31/128) and a dMMR phenotype was found in 16.8% (*n* = 22/131). Among the 480 molecular tests in Poitou-Charentes incident cases of CRC, 83.5% (*n* = 401) were performed in the platform of Poitiers and 16.5% (*n* = 79) outside the region.

The average time to obtain results of molecular tests, defined by the interval between the date of histological sampling and the date of molecular test results available in the platform, was 30.6 days for *KRAS* testing, 36.3 days for *BRAF* testing and 41.3 days for MSI testing.

### *KRAS* testing

*KRAS* molecular testing was carried out in 35.1% (*n* = 445/1269), including 65.5% (*n* = 190/290) metastatic and 26.1% (*n* = 255/979) non-metastatic CRC patients. *KRAS* molecular testing was mainly requested by pathologists (*n* = 174, 39.1%), surgeons (*n* = 105, 23.6%) and oncologists (*n* = 84, 18.9%) (Table 2). Among mCRC patients, 68.6% (*n* = 199/290) received chemotherapy and among them 83.9% (*n* = 167/199) had *KRAS* molecular testing.

**Table 2:** Specialty of physicians who order molecular testing

	*KRAS* (*n* = 445)	*BRAF* (*n* = 133)	MSI (*n* = 138)
Pathologists	174 (39.1%)	38 (28.6%)	34 (24.6%)
Surgeons	105 (23.6%)	22 (16.5%)	21 (15.2%)
Oncologists	84 (18.9%)	37 (27.8%)	43 (31.2%)
Gastroenterologists	9 (2.0%)	1 (0.7%)	1 (0.7%)
Others	4 (0.9%)	7 (5.3%)	5 (3.6%)
Non communicated/unknown	69 (15.5%)	28 (21.1%)	34 (24.6%)

Among overall cohort, age at diagnosis, site of primary tumor, stage at diagnosis, geographical area of primary treatment and status of the center were the factors related to *KRAS* testing (data not shown). Secondly, analyses of metastatic and non-metastatic CRCs were performed separately, given that *KRAS* testing is recommended only in cases of mCRC.

Among mCRC patients, in multivariate analysis, age at diagnosis (<75 years; *p* < 0.0001), site of primary tumor (left colon; *p* = 0.006) and geographical area of primary treatment (*p* = 0.01) were factors related to *KRAS* molecular testing (Table 3). All mCRC patients treated with an anti-EGFR mAbs had *KRAS* molecular testing (*n* = 42). Among *KRAS* wild-type mCRC (*n* = 101), 40.6% were treated with anti-EGFR mAbs. More than half of *KRAS* molecular testing for mCRC patients was requested by pathologists (*n* = 60, 31.6%) and oncologists (*n* = 51, 26.3%).

**Table 3:** Factors influencing *KRAS* testing in metastatic CRC patients

	*KRAS* testing *n* = 190/290 (65.5%)	Univariate analysis *P-Value*	Multivariate analysis		
			Odds ratio	95% CI	*P-Value*
Age (years)		< 0.0001			< 0.0001
> 75	42 / 107 (39.3%)		1	Ref	
60–75	97 / 126 (77.0%)		4.72	2.54–8.77	
< 60	51 / 57 (89.5%)		10.78	4.07–28.50	
Sex		0.016			0.33
Women	82 / 140 (58.6%)		1	Ref	
Men	108 / 150 (72.0%)		1.34	0.75–2.41	
Site of the primary tumour		0.0011			0.006
Rectum	35 / 64 (54.7%)		1	Ref	
Right colon	51 / 90 (56.7%)		1.44	0.67–3.07	
Left colon	104 / 136 (76.5%)		3.09	1.48–6.45	
Tumour grade (MD = 40)		0.52			
Well	63 / 89 (70.8%)				
Moderate	83 / 131 (63.4%)				
Poor	20 / 30 (66.7%)				
Geographical area of primary treatment (MD = 2)		0.0001			0.010
Charente-Maritime	42 / 87 (48.3%)		1	Ref	
Charente	32 / 53 (60.4%)		1.99	0.89–4.46	
Deux-Sèvres	37 / 45 (82.2%)		4.64	1.77–12.18	
Vienne	61 / 79 (77.2%)		2.88	1.36–6.13	
Outside the region	17 / 24 (70.8%)		2.02	0.67–6.12	
Status of the center (MD = 2)		0.026^a^			
Public Hospital	60 / 83 (72.3%)		–	–	–
Private hospital	83 / 143 (58.0%)				
University hospital	46 / 62 (74.2%)				

*95% CI* 95% confidence interval, *MD* missing data, *Ref* reference

^a^Not retained in the final multivariate model

Among non-metastatic CRC patients, in multivariate analysis, age at diagnosis (<75 years; *p* < 0.0001), site of primary tumor (right colon; *p* = 0.026), stage at diagnosis (stage II and III; p < 0.0001), geographical area of primary treatment (p < 0.0001) and status of the center (private hospital; p < 0.0001) were factors related to *KRAS* molecular testing (Table 4). *KRAS* molecular testing for non-metastatic CRC patients was mainly requested by pathologists (*n* = 114, 44.7%) and surgeons (*n* = 72, 28.2%).

**Table 4:** Factors influencing *KRAS* testing in non-metastatic CRC patients

	*KRAS* testing *N* = 255/979 (26.1%)	Univariate analysis *P-Value*	Multivariate analysis		
			Odds ratio	95% CI	*P-Value*
Age (years)		0.0004			< 0.0001
> 75	90 / 446 (20.2%)		1	Ref	
60–75	123 / 382 (32.2%)		2.69	1.83–3.95	
< 60	42 / 151 (27.8%)		2.26	1.34–3.81	
Sex		0.25			0.15
Women	133 / 541 (24.6%)		1	Ref	
Men	122 / 438 (27.9%)		0.77	0.55–1.09	
Site of the primary tumour		0.010			0.026
Rectum	47 / 245 (19.2%)		1	Ref	
Right colon	105 / 347 (30.3%)		1.95	1.20–3.16	
Left colon	103 / 387 (26.6%)		1.47	0.92–2.35	
TNM stage		< 0.0001			< 0.0001
I	21 / 219 (9.6%)		1	Ref	
II	100 / 380 (26.3%)		5.24	2.98–9.21	
III	134 / 380 (35.3%)		9.62	5.47–16.90	
Tumour grade (MD = 133)		0.0082^a^			
Well	114 / 347 (32.8%)		–	–	–
Moderate	103 / 445 (23.2%)				
Poor	17 / 54 (31.5%)				
Geographical area of primary treatment (MD = 2)		< 0.0001			< 0.0001
Charente-Maritime	63 / 305 (20.7%)		1	Ref	
Charente	13 / 173 (7.5%)		0.19	0.10–0.37	
Deux-Sèvres	75 / 182 (41.2%)		3.74	2.35–5.95	
Vienne	94 / 222 (42.3%)		3.24	1.95–5.39	
Outside the region	10 / 95 (10.5%)		0.36	0.16–0.79	
Status of the center (MD = 2)		< 0.0001			< 0.0001
Public Hospital	77 / 408 (18.9%)		1	Ref	
Private hospital	149 / 429 (34.7%)		4.18	2.74–6.37	
University hospital	29 / 140 (20.7%)		0.88	0.44–1.75	

*95% CI* 95% confidence interval, *NA* not available, *MD* missing data, *Ref* reference

^a^Not retained in the final multivariate model

### *BRAF* testing

*BRAF* molecular testing was performed in 10.5% (*n* = 133/1269), including 18.6% (*n* = 54/290) metastatic and 8.1% (*n* = 79/979) non-metastatic CRC patients. *BRAF* molecular testing was mainly requested by pathologists (*n* = 38, 28.6%), oncologists (*n* = 37, 27.8%) and surgeons (*n* = 22, 16.5%). *BRAF* molecular testing was contemporary to *KRAS* molecular testing for 92.5% of CRC patients (*n* = 123/133), of whom 93.5% (*n* = 115/123) were *KRAS* WT. Among the 101 *KRAS* WT mCRC patients, 47.5% (*n* = 48) had *BRAF* testing. Considering that *BRAF* testing should be performed in case of *KRAS* WT status, the factors associated with *BRAF* testing were not detailed as they were in fact similar to those for *KRAS* testing.

### MSI testing

MSI molecular testing was performed in 10.9% (*n* = 138/1269), 39.4% (*n* = 82/208) in patients under 60 years and 5.3% (*n* = 56/1061) in patients over 60 years. MSI molecular testing was mainly requested by oncologists (*n* = 43, 31.2%) and pathologists (*n* = 34, 24.6%). Among the 138 patients with MSI testing, 58.0% (*n* = 80/138) had no *BRAF* testing. There was no significant difference in proportion of MSI testing between *BRAF*-mutated and *BRAF* WT CRC, respectively 38.7% (*n* = 12/31) and 43.3% (*n* = 42/97) (*p* = 0.65).

In multivariate analysis, age at diagnosis (<75 years; *p* < 0.0001), stage at diagnosis (stage II, III and IV; *p* < 0.0001) and geographical area of primary treatment (*p* < 0.0001) were factors related to MSI testing (Table 5). Among patients under 60 years old, 39.4% (*n* = 82/208) had MSI testing and 11.5% had an oncogenetic consultation (*n* = 24/208). Overall, among the 22 patients with dMMR CRC, we identified 6 *BRAF* wild-type CRCs (27.3%), 9 *BRAF*-mutated CRCs (40.9%) and 7 without *BRAF* testing (31.8%). Among patients with dMMR CRC and *BRAF* wild-type status or no *BRAF* testing, 61.5% had an oncogenetic consultation (*n* = 8/13).

**Table 5:** Factors influencing MSI testing in all CRC patients

	MSI testing *N* = 138/1269 (10.9%)	Univariate analysis *P-Value*	Multivariate analysis		
			Odds ratio	IC 95%	*P-Value*
Age (years)		< 0.0001			< 0.0001
> 75	10 / 553 (1.8%)		1	Ref	
60–75	46 / 508 (9.1%)		5.77	2.80–11.89	
< 60	82 / 208 (39.4%)		59.6	27.82–127.85	
Sex		0.88			0.21
Women	62 / 578 (10.7%)		1	Ref	
Men	76 / 691 (11.0%)		0.75	0.47–1.18	
Site of the primary tumour		0.53			
Rectum	31 / 309 (10.0%)				
Right colon	44 / 437 (10.1%)				
Left colon	63 / 523 (12.1%)				
TNM stage		< 0.001			0.0001
I	6 / 219 (2.7%)		1	Ref	
II	37 / 380 (9.7%)		8.62	3.17–23.41	
III	49 / 380 (12.9%)		7.96	3.00–21.12	
IV	46 / 290 (15.9%)		8.79	3.29–23.48	
Tumour grade (MD = 173)		0.34			
Well	55 / 436 (12.6%)				
Moderate	56 / 576 (9.7%)				
Poor	9 / 84 (10.7%)				
Geographical area of primary treatment (MD = 4)		< 0.0001			< 0.0001
Charente-Maritime	3 / 392 (0.8%)		1	Ref	
Charente	21 / 226 (9.3%)		17.08	4.78–61.00	
Deux-Sèvres	19 / 227 (8.4%)		13.98	3.89–50.24	
Vienne	80 / 301 (26.6%)		69.59	20.51–236.01	
Outside the region	15 / 119 (12.6%)		13.53	3.64–50.29	
Status of the center (MD = 4)		< 0.0001^a^			
Public Hospital	36 / 551 (6.5%)		–	–	–
Private hospital	47 / 512 (9.2%)				
University hospital	55 / 202 (27.2%)				

*95% CI* 95% confidence interval, *NA* not available, *MD* missing data, *Ref* reference

^a^Not retained in the final multivariate model

## Discussion

Our study is the first one to simultaneously evaluate three molecular testing procedures (*KRAS, BRAF* and MSI) in CRC. Rates for these molecular testing procedures were systematically linked to age at CRC diagnosis, site of primary tumour, stage at diagnosis, geographical area of primary treatment and status of the center.

*KRAS* testing was performed in 35.1% of CRCs and as expected was more frequent in patients with a metastatic disease (65.5%). Although *KRAS* status is required for the anti-EGFR mAbs used in mCRC, there are few data on *KRAS* testing rates. In a French retrospective study conducted in 2011 81.1% of mCRCs had *KRAS* testing [9] which is higher as compared our work. However, there are selection biases in Lièvre et al. study since patient recruitment was based on physician willingness. Finally, our rate is in accordance with that found in a large retrospective study published in 2011 concerning Europe, Latin America and Asia (69%) [10]. Moreover, in our study when limited to mCRC patients receiving first-line chemotherapy, *KRAS* molecular testing rate was higher (83.9%).

Among mCRC patients, in multivariate analysis young age at diagnosis, primary tumor located in left colon and geographical area of primary treatment were factors related to *KRAS* molecular testing. Frequent *KRAS* testing in young patients is probably explained by more “aggressive” treatment strategies in these patients, particularly anti-EGFR mAbs used. We have no explanation as to why *KRAS* testing was more frequent for left-sided tumors. *KRAS* testing was also significantly more frequent in the Vienne and Deux-Sèvres departments. In the Poitou-Charentes region there is only one university hospital located in the Vienne department. We can suppose that the higher rate of *KRAS* testing in Vienne department was linked to university hospital research programs and easier access to molecular testing. We observed that molecular testing procedures were mainly requested by pathologists and oncologists. An earlier request by gastroenterologists on initial biopsies should be encouraged to allow the availability of molecular tests results during the first oncological consultation in order to quickly define the optimal treatment for mCRC (RAS status and anti-EGFR treatment).

Our work showed that 26.1% of non-metastatic CRC cases had *KRAS* testing. The rate in the USA population is 5% [11]. Younger age, higher stage at diagnosis, geographical area of primary treatment and status of the center were factors related to *KRAS* molecular testing in non-metastatic CRCs. We can suppose that it was conducted at the request of the clinician to quickly begin appropriate treatment in the event of development of metachronous metastases, especially in stage III patients. In addition, for some pathologists it was easier to address pathological samples to a molecular cancer genetics platform at the time of the first pathological examination rather than later, when the tumor blocs were archived.

To our knowledge there has been no previous study evaluating *BRAF* testing rates in CRC cases. In our study the rate of *BRAF* testing was 10.5% and the factors influencing *BRAF* testing are similar to those influencing *KRAS* testing. The rate of *BRAF*-mutated CRC (24.2%) was high as compared with the literature (approximately 12%) [12, 13]. *BRAF* testing was mostly performed directly by molecular cancer genetic platform in patients with *KRAS* wild-type CRC since the two mutations are mutually exclusive. This point explains the high rate of *BRAF*-mutated CRC since only *KRAS* WT CRCs were analyzed for *BRAF*.

Concerning MSI testing, the rate seems low (10.8%) but the dMMR CRC rate is in accordance with literature data [14, 15]. To our knowledge this is the first study that analyzing factors related to MSI testing rates. Like *KRAS* testing, MSI testing was associated in multivariate analysis with young age, higher tumor stage and geographical area of primary treatment. French guidelines recommended MSI testing for patients under 60 years old and/or *BRAF*-mutated CRC. Consequently, MSI testing was performed directly by the molecular cancer genetics platforms for patients under 60 years old and/or *BRAF*-mutated CRC when there was *KRAS*/*BRAF* testing. These points explain how it is that the factors influencing MSI testing are close to those influencing *KRAS*/*BRAF* testing.

Our study highlights the fact that guidelines for LS screening are not well-respected. Only 39.4% of CRC patients under 60 years old had MSI testing and some dMMR CRCs (31.8%) did not have *BRAF* testing to identify sporadic cases. Finally, most patients with a suspicion of LS (dMMR CRC with no *BRAF* mutation) did not have an oncogenetic consultation (38.5%). We were not able to determine if this was due to patient refusal or if patients had not been addressed to an oncogenetic consultant by their referring physician.

The average time to obtain results of *KRAS* tests in our study was 30.6 days (between histological sampling and the date when the molecular test results were available in the platform). Lièvre et al.*,* calculated the median delay between physician prescription and reception of the results as 23.6 ± 28.2 days, a delay somewhat shorter because measured differently [9]. In addition, in contrast to the Lièvre et al. study, our study is reflective of real life and exhaustive. To our knowledge, no previous study evaluated delays in *BRAF* and MSI testing.

The main strength of our study resides in the crossing of two reliable and exhaustive data banks, GCRPC and INCa molecular cancer genetics platforms. If none of the previous studies evaluating *KRAS* testing are as exhaustive, it is because they were based on incomplete database and/or on questionnaires sent to volunteer physicians. The main limitation of our work is the difficulty in extrapolating its results to other countries since CRC molecular tests are dependent on physicians’ and pathologists’ clinical practices. It is noteworthy that we accessed the molecular testing rates in 2010 since there is a delay of at least 2 years before obtaining high-quality CRC data from the GCRPC, a delay justified by the data collection process and the application of standards and requirements during case registration. Moreover, it is challenging to retrieve reliable and retrospective information on life-style and family history, but it would be interesting to complete this evaluation by including CCR risk factors which probably influences the choice of the clinician for ordering molecular testing. Finally, factors influencing these molecular testing procedures are relevant for countries which already performed these tests but also those who are implementing these tests in order to allow an optimal use, especially RAS testing for anti-EGFR therapy used in mCRC.

## Conclusion

To conclude, this study is the first to provide a robust and exhaustive overview of molecular testing in CRC. As expected, we note a high level of *KRAS* testing in mCRC but also significant level in stage III CRC, which was probably undertaken in order to have *KRAS* results for patients with a high risk of disease recurrence. Moreover, MSI testing rate is low and not in accordance with French guidelines, which recommend systematic testing before the age of 60. In addition, these results highlighted on which factors it is possible to act to improve the molecular testing procedures essential to management of CRC patients, particularly MSI testing.

## Abbreviations

CI: Confidence interval
CRC: Colorectal cancer
dMMR: Deficient DNA mismatch repair
GCRPC: General cancer registry in the poitou-charentes region
LS: Lynch syndrome
MSI: Microsatellite instability
OR: Odds ratios
WT: Wild-type
